# British South Asian Patients’ Perspectives on the Relevance and Acceptability of Mobile Health Text Messaging to Support Medication Adherence for Type 2 Diabetes: Qualitative Study

**DOI:** 10.2196/15789

**Published:** 2020-04-20

**Authors:** Suman Prinjha, Ignacio Ricci-Cabello, Nikki Newhouse, Andrew Farmer

**Affiliations:** 1 Nuffield Department of Primary Care Health Sciences University of Oxford Oxford United Kingdom; 2 Balearic Islands Health Services Primary Care Research Unit of Mallorca Palma de Mallorca Illes Balears Spain; 3 Health Research Institute of the Balearic Islands (IdISBa) Palma de Mallorca Illes Balears Spain; 4 Ciber de Epidemiologia y Salud Pública (CIBERESP) Madrid Spain

**Keywords:** type 2 diabetes, South Asians, text messages, self-management, medication adherence, mobile health, mHealth, eHealth

## Abstract

**Background:**

The prevalence of type 2 diabetes (T2D) is greater in South Asian populations and health outcomes are poorer compared with other ethnic groups. British South Asians are up to six times more likely to have T2D than the general population, to develop the condition at a younger age, and to experience diabetes-related complications. Interventions to support people in managing their condition can potentially reduce debilitating complications. Evidence to support the use of digital devices in T2D management, including mobile phones, has shown positive impacts on glycemic control. There is increasing recognition that health interventions that are culturally adapted to the needs of specific groups are more likely to be relevant and acceptable, but evidence to support the effectiveness of adapted interventions is limited and inconclusive.

**Objective:**

This formative study aimed to explore the perceptions and views of British South Asian patients with T2D on mobile health SMS text messaging to support medication adherence, aimed at the general UK population.

**Methods:**

Eight exploratory focus groups were conducted in Leicester, the United Kingdom, between September 2017 and March 2018. A diverse sample of 67 adults took part.

**Results:**

British South Asian people with T2D who use digital devices, including mobile phones, felt that short messages to support medication adherence would be acceptable and relevant, but they also wanted messages that would support other aspects of self-management too. Participants were particularly interested in content that met their information needs, including information about South Asian foods, commonly used herbs and spices, natural and herbal approaches used in the United Kingdom and in South Asia, and religious fasting. Short messages delivered in English were perceived to be acceptable, often because family members could translate for those unable to read or understand the messages. Suggestions to support patients unable to understand short messages in English included having them available in different formats, and disseminated in face-to-face groups for those who did not use digital devices.

**Conclusions:**

Exploring the views of British South Asian patients about SMS text messaging aimed at the general UK population is important in maximizing the potential of such an intervention. For such a digital system to meet the needs of UK South Asian populations, it may also have to include culturally relevant messages sent to those who opt to receive them. It is equally important to consider how to disseminate message content to patients who do not use digital devices to help reduce health inequalities.

## Introduction

### Digital Health Interventions to Support Patients With Type 2 Diabetes

The prevalence of type 2 diabetes (T2D) is increasing globally, representing a serious clinical and financial challenge [1]. Diabetes prevalence is greater in South Asian populations and health outcomes are poorer [2]. South Asians account for approximately one-fifth of the global population. T2D occurs at 50% higher rates in South Asian patients compared with the general population [3], developing 5 to 10 years earlier, and is one of the main causes of premature death in this population [4]. South Asians constitute the largest minority ethnic group in the United Kingdom, around 7% of the total British population [5]. UK South Asians are up to six times more likely to have T2D than the general population, to develop the condition at a younger age, and to experience diabetes-related complications [6].

Effective diabetes self-management (DSM)—healthy diet, physical activity, and medication adherence—is associated with improved glycemic control [7], leading to a reduction in complications and mortality [8]. Interventions to support people in effectively self-managing their condition can potentially reduce costly, debilitating complications [9,10]. Interventions to support medication adherence can lead to improved health outcomes [11]. Digital health technologies have the potential to deliver low-cost interventions aimed at supporting healthier lifestyles [12] and disease self-management [13,14]. The number of mobile phone users surpassed 2 billion in 2016 and is expected to increase to 2.86 billion by 2020 [15]. In the United Kingdom, 73% of adults accessed the internet through a smartphone or mobile device in 2017 [16]. The growing evidence supporting the use of mobile phone–based technologies in T2D management, including SMS text messages, has shown positive impacts on glycemic control [17] and health care costs [18].

### Cultural Adaptation of Health Interventions

Research into the cultural adaptation of health promotion interventions, including SMS text messaging interventions, is growing steadily, with an increasing recognition that lifestyle and behavior change interventions that are culturally adapted to the needs of specific groups are more likely to be effective [19-21]. Cultural adaptation involves grounding interventions in the lived experience of end users, taking account of language, cultural patterns, and values [22,23]. Mobile health (mHealth) interventions have been culturally adapted and piloted or trialed in several countries, including in New Zealand to support healthy lifestyles in Māori and Pasifika communities [24,25]; maternal health in Māori, Pacific, Asian, and South Asian families [26]; and smoking cessation [27]. Interventions aimed at the general population tend to be less effective for Māori and Pasifika communities [28], possibly contributing to increased health inequalities [29]. These interventions have often been designed with little input from these communities, have lacked tailoring to cultural needs, and have had poor uptake [24].

The few culturally adapted complex interventions for UK South Asian populations with T2D have been lifestyle modification interventions (diet-, weight-, and physical activity–related) rather than digital health interventions [30,31]. However, evidence to support the effectiveness of these interventions is inconclusive in demonstrating sufficient health-related gain to make such interventions cost effective [32,33]. Head-to-head comparisons of adapted and nonadapted interventions are rare [34], though the use of adaptations has been documented to increase process outcomes such as acceptability, uptake, satisfaction, and retention [35].

Previous research into DSM in British South Asian populations has explored perceptions and experiences of taking prescribed diabetes medications and traditional medicines [36,37], and barriers and facilitators to diet management [38,39] and physical activity [40]. Patients’ perceptions of oral diabetes medications are complex and ambivalent, with good patient-provider communication and an understanding of the cultural factors that inform beliefs and practices reported as key to improving medication adherence [41]. Diet-management has been highlighted to be the most difficult aspect of living with diabetes, with recommendations including designing interventions that involve family members as well as patients [42].

At present, there is no published research into digital health interventions for UK South Asian patients with T2D, or the cultural adaptation of digital health interventions aimed at the general population. The EuroDHYAN study currently underway in the United Kingdom includes a pilot study of SMS text messages for T2D prevention in women of Pakistani origin in Scotland [43]. We know little about how best to adapt interventions to meet the needs of South Asian migrant populations with T2D [44]. This formative research aimed to explore the perceptions and views of British South Asian patients with T2D on mHealth SMS text messaging to support medication adherence.

### Study Design

Recent research into digital health intervention development highlights the importance of an iterative, multidisciplinary approach in which the end user is placed at the heart of the system and the technology is grounded in user wants and needs [45-47]. This is increasingly seen as crucial to person-centered or person-based intervention development [48-50]. Research examining SMS text messaging interventions, in particular, has highlighted the use of multiple methodologies in intervention development, including qualitative research in the planning stage to gather information from relevant stakeholders and to inform decisions about message frequency, timing, and level of tailoring [51].

Exploratory focus groups were conducted with a range of South Asian communities in Leicester, one of the most ethnically diverse cities in England [52]. The focus group study was part of a larger project, the SuMMiT-D (Support Through Mobile Messaging and Digital Health Technology for Diabetes) study, which aimed to explore supporting people with T2D in effective use of their medicine through a system comprising digital health technology integrated with clinical care, including use of SMS text messages [53]. Messages were developed using a taxonomy of behavior change techniques to identify ways of structuring a wide range of messages derived from patients, health care professionals, and health promotion literature [54]. An extensive library of messages was developed by a meeting of psychologists and health care professionals, checked for fidelity to behavior change techniques and checked for acceptability to patients [55]. The focus group study was a collaboration between the University of Oxford and the Centre for Black and Minority Ethnic Health (CBMEH), University of Leicester.

## Methods

### Ethical Approval

Ethical approval was obtained from the University of Oxford Central University Research Ethics Committee (Ref R50751/RE001).

### Recruitment and Participants

British South Asian populations include first, second, and third generation people of Indian, Pakistani, Bangladeshi, and Sri Lankan descent. Participants were purposively sampled to include a broad range of views [56], reflecting the heterogeneity within and across UK South Asian communities, including people of different age groups, educational and occupational backgrounds, fluency in English, place of birth, and time since diagnosis. We also aimed to include users and nonusers of digital devices. Adults with T2D were recruited from community centers, places of worship, and a South Asian women’s center in Leicester, with the support of the CBMEH who had strong links with local community organizations. Potential participants were informed about the focus groups verbally and with written information in English by a CBMEH project support worker and by community center managers.

### Focus Group Discussions

We conducted eight exploratory focus groups between September 2017 and March 2018. Discussions were held in venues that participants were familiar with and could easily access, such as community centers. Each group met once, and the discussions lasted between 1.5 and 2 hours. The focus groups were facilitated by SP, a qualitative researcher, with cofacilitators from the CBMEH. Before the start of the focus group, the facilitators briefly introduced the study and discussed informed consent and confidentiality in the preferred language of participants. All participants gave signed consent. A topic guide was used to facilitate discussion, informed by the multidisciplinary literature on British South Asian experiences of T2D (Textbox 1). A detailed description of our methods is discussed elsewhere [57].

**Figure 1:**
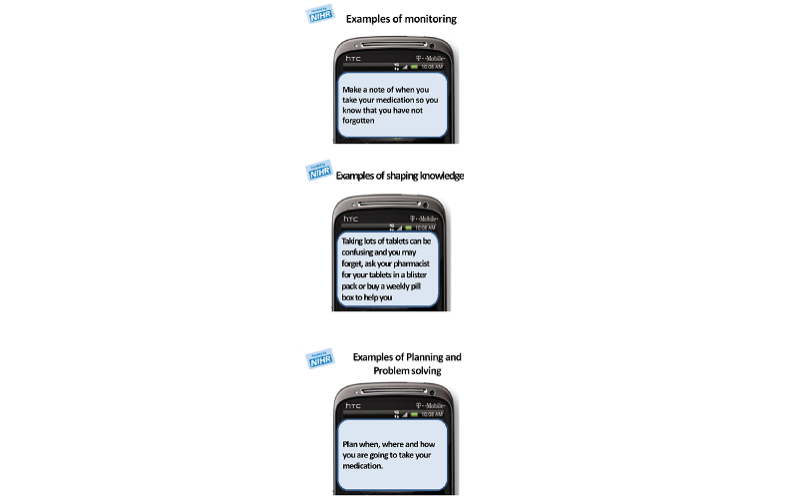
Examples of messages from the Support Through Mobile Messaging and Digital Health Technology for Diabetes system.

The focus groups aimed to explore South Asian perspectives of DSM, engagement with digital devices, support needs, and whether an SMS could help them to manage their condition. Examples of SMS text messages from the SuMMiT-D program were also discussed (Figure 1). Data collection continued until no new themes relating to the system were identified.

**Focus group topic guide.**

Part 1: Challenges of living with type 2 diabetes and self-management

What do you find most difficult about living with diabetes?

- *Medication*
  - When is it difficult or challenging to take the tablets as recommended by the doctor?
  - When do you find it easiest to stick to a routine with your medications?
  - Do you use any herbal or non-Western approaches to help you with your diabetes?
- *Physical activity*
  - When is it difficult or challenging to exercise, go for a walk, or keep fit and healthy physically?
  - When do you find it easiest to stick to a routine with exercise and keeping fit and well?
- *Diet*
  - When is it difficult or challenging to eat healthily?
  - When do you find it easiest to stick to a routine with healthy eating?
  - Explore everyday diet and diet during festivities/family gatherings/during fasting.
  - What sorts of things do you do to help you to look after your diabetes and to stay as healthy as you can?
  - What might help you to manage your diabetes?

Part 2: Views on brief digital messaging system

- If we were going to design a new brief messaging system (show images), what kind of system and messages would help you most?
- Explore thoughts and feelings about the system and whether participants use mobile phones/digital devices.
- What do you think a system like this could include?
- What would make a system like this acceptable to you? What would make a system like this unacceptable to you?
- *System messages*
  - Do you have any problems with understanding any of the messages?
  - Explore suggestions to improve the messages so they can be more easily understood.
  - Were the messages clear and easy to understand?
  - How would you change the messages (use of language, tone, personalization)?
  - Do you think it is helpful to receive messages in English or to translate these messages? What are your thoughts about translated messages?
  - Do you think the proposed messages would help you personally to take your diabetes medication?
  - Who would you want the messages to come from? (National Health Service? General Practitioner? Pharmacist? Researchers?)
  - How often would it be helpful to receive messages like these?
  - Do you think this system would help you manage your health and diabetes better?
  - Would you sign up to a system like this? Explore reasons for and against.

### Data Analysis

Discussions were conducted in Punjabi, Bengali, Sylheti, Urdu, Hindi, and English. They were audio-recorded, and translated and transcribed verbatim by SP or a professional transcriber. SP conducted the initial thematic analysis [58], coding inductively for main themes using a qualitative software package (NVivo, QSR International Pty Ltd, Melbourne, Australia, Version 12, 2018) [59]. Transcripts were then independently coded by NN. All four research team members (SP, RC, NN, and AF) discussed the data and themes, and finalized themes using a consensus process. Discrepancies were resolved and agreed by consensus.

## Results

A total of 67 participants (including four carers) were recruited from some of the largest South Asian communities in the United Kingdom: Indian Punjabi Sikh, Pakistani Muslim, Indian Gujarati Hindu, Bangladeshi Muslim, and Indian Gujarati Muslim. Participants ranged in age from 18 to 84 years and included first- and second-generation South Asians. Four groups were mixed and four were single sex; groups ranged in size from n=5 to n=12 (Table 1).

Users and nonusers of digital devices discussed a range of challenges in terms of DSM and suggested a number of potential ways that SMS messages could help. Views ranged from those who felt that such a system would be a very useful addition to supporting existing self-management endeavors to those who stated that they would not use SMSs as they rarely used digital devices at all, including mobile phones. Users of digital devices discussed not only their own needs and preferences relating to the system but also their views and perceptions of what would be helpful for nonusers in their families and communities. Likewise, nonusers discussed their preferences and how family members who were users of digital technologies could help them. Five main themes were identified from the data, relating to system content and usability: (1) message content and design features, (2) language preferences, (3) family involvement, (4) different digital formats for different groups, and (5) face-to-face groups for those who do not use digital devices (Multimedia Appendix 1).

**Table 1:** Focus group composition and participant demographics (N=67).

Language/cultural group	Participants^a^, n	Male^b^, n	Female^c^, n	Age range (years)	Country of birth (number of participants)
Punjabi Sikh men and women	11	5	6	47-78	India (11)
Bangladeshi Muslim men	11	11	0	41-81	Bangladesh (10) United Kingdom (1)
Pakistani Muslim men and women	7	3	4	39-66	Pakistan (3) India (1) Bangladesh (1) Malawi (1) Mozambique (1)
Gujarati Hindu men and women	8	4	4	56-84	India (4) Kenya (2) Uganda (1) Trinidad (1)
South Asian women	12	0	12	18-71	Bangladesh (3) Pakistan (3) India (2) Sri Lanka (1) Uganda (1) Malawi (1) United Kingdom (1)
Bangladeshi Muslim women	7	0	7	34-45	Bangladesh (7)
Gujarati Muslim men	5	5	0	50-75	India (4) Malawi (1)
Younger people aged 18-45 years	6	1	5	28-47	Bangladesh (6)

^a^Total=67.

^b^Total=29.

^c^Total=38.

### Message Content and Design Features

Participants who used mobile phones or other digital devices were positive about receiving short messages about diabetes medication and the importance of taking medicines. However, they felt strongly that a messaging service should help support all aspects of self-management and not only medication adherence (Textbox 2).

The content of messages was a common theme, with many people discussing the kinds of messages that they would like to receive. Medication-related messages that they wanted included: information about diabetes symptoms, side effects, the risks associated with not taking medicines, the long-term effects of medications on kidney health, whether tablets should be taken before or after food, routine blood tests, and how to improve glycemic control. Participants expressed strong interest in messages that addressed unmet information needs, particularly about diet and physical activity, including messages about South Asian foods, portion sizes, and fasting. Participants also expressed a need for information about stress and stress management, natural and complementary approaches used in the United Kingdom and South Asia, and “reversing” diabetes:

In my opinion some kind of a diet plan should be introduced showing, for example, if you eat this amount of food in the morning, and in the afternoon one small bowl of lentils and one chapatti for example... If there was a diet plan, we could look at it and follow it. That would help.... When you go to see your doctor, they could show you that this is a plate and this is the portion size that should fill this plate....But these diet plans and portion sizes don’t exist for Punjabi diets. They don’t exist for our diets and foods.

53-year-old Punjabi Sikh female

**Patients’ preferences for message content.**

*General information*

- *Medication-related information*
  - Diabetes symptoms
  - Side effects
  - Risks associated with not taking medicines
  - Long-term effects of medications on kidney health
  - Whether tablets should be taken before or after food
  - Routine blood tests
  - New medications for diabetes
  - How to improve glycemic control
  - Why some people have to take tablets as well as insulin to manage diabetes?
- *Information about diet*
  - Healthy eating
  - Portion sizes
  - Sugar content in foods including fruits (eg, mangoes and bananas)
  - Dietary guidelines
  - Effects of commonly consumed foods on blood sugar levels
- *Information about physical activity*
  - Recommendations for walking
  - Exercises for people unable to walk because of disability (eg, exercises they could do while sitting)
- *Other information to support self-management*
  - Stress and stress management
  - “Reversing” diabetes
  - Information about current research
  - New research findings
  - Details of local diabetes-related events such as talks and discussions

*Information relating to South Asian culture and self-management*

- Healthy South Asian diets
- Effects of South Asian foods on blood sugar levels (eg, rice, chapatti, and different types of chapatti flour)
- South Asian diet plans and portion sizes
- Herbs and spices that may have health benefits (eg, turmeric, cinnamon, fennel, and neem)
- Health benefits of honey and dates
- Natural and complementary approaches used in the United Kingdom and South Asia
- Fasting and safe medicine taking
- Medicine taking when traveling to South Asia
- Local women-only gyms and swimming classes
- Details of local diabetes-related events for South Asian communities

Participants also discussed system design features, with views varying vastly and little consensus. In terms of the frequency of messages, views ranged from participants who preferred to have messages daily to those who wanted them weekly, fortnightly, or monthly. Views on the personalization of messages varied between those who were in favor of having their first name being used to those who did not want this. Messages sent by general practitioners (GPs) or researchers were seen to be credible and trustworthy:

If we're going to get too many of these messages, people are just going to ignore them...[...]

58-year-old man, Gujarati Hindu focus group

That was what I was suggesting, that if you get too many of them you don’t even look at it. Yeah, [then] they're a sheer waste of effort and time.

69-year-old man, Gujarati Hindu focus group

### Language Preferences

Participants felt that messages in English would be acceptable as “everyone understands English” and that those who spoke no or little English could receive help to translate messages from their children, often providing examples of a son or daughter who could help in these situations. Several who preferred messages in English noted that the written version of some South Asian languages might be too formal and difficult for most people to understand, that some dialects have no written form, and that many people who spoke a South Asian language could not always read or write it. Some younger participants who spoke little English (and had recently migrated to the United Kingdom) believed that receiving SMS text messages in English might help them to improve their English and that Google Translate could be used when needed:

If it is translated into Bengali we will not learn English as we have an alternative.

34-year-old Bangladeshi Muslim woman

This is a very good point actually i.e. if it is said in our respective language, then the motivation to learn English would decrease because we will find whatever it is we want, so we probably won’t learn.

47-year-old Bangladeshi Muslim man

The translation of short messages into South Asian languages was seen as a possible option to consider only if resources were available. Some participants felt that while multi-language options would provide choice and should perhaps be ideally available, these options were unnecessary given how brief the messages were and quick and easy for family members to translate for patients unable to read them:

English is okay, it’s just information [laughs]. [If you don’t read English] you’d show someone else briefly to tell you that’s what it says about diabetes. It’s easy, and that’s it, finish.

50-year-old Gujarati Muslim man

### Family Involvement

Family involvement, key for participants—particularly those unable to communicate well or fluently in English—was a theme discussed in all focus groups. This included the role of young and adult children in helping patients to self-care, and the support of spouses and carers. Participants often discussed how family members (eg, spouses) reminded the patient to take medications, cooked healthier meals since their diagnosis, and made or attended GP appointments with them. One participant, a 66-year-old Pakistani Muslim man, noted that his wife reminded him to take his medications, helped him watch his diet, and that messages should be sent to her phone rather than his. Another stated that messages for his father’s physiotherapy appointments were already being sent to his phone because his father was unable to read them. He, like others, felt that brief messages about T2D should be sent to family members when the patient did not use or have a mobile phone, or was unable to read English, as family members could translate or explain messages to the patient:

For instance, my dad, he has physiotherapy appointments. My dad had a fall, but sending a text message to my dad is not good. Okay. So I have given them my number. So I get the message...I take him for his physiotherapy appointment.

47-year-old Bangladeshi Muslim man

### Different Digital Formats for Different Groups

Although younger people generally engaged more readily with digital devices than their older counterparts, participants felt that messages in different formats could enable all members of their communities to benefit from text messages, noting the diversity within families and communities in terms of education and fluency in English. Discussions around different formats for different groups included SMS text messages in English for those who could read and understand them, audio messages in English for those who could understand but not read English, possibly audio messages in South Asian languages for those who required this, and illustrations or images to help those unable to read English or a South Asian language or dialect.

M1: *What should we do for those who are not educated and cannot read or write?*

M7: *We can send messages via Whatsapp.*

M1: *Do you mean send images via Whatsapp?*

M7: *Images or recorded messages [...]*

M6: *Images, animations.*

[M1: 47-year-old; M7: 41-year-old; M6: 46-year-old; Bangladeshi Muslim men’s focus group]

### Face-to-Face Groups for Those Who Do Not Use Digital Devices

Information provision was seen as essential for good DSM, but participants felt that it was not reaching all sections of their communities. They emphasized that not everyone, particularly older patients, had a mobile phone: “Older people don’t even have mobile phones,” carry them on their person, or charge them regularly. Two suggestions were offered in these cases: (1) sending messages to other family members who were involved in their care and (2) face-to-face meetings as an opportunity for people to share information and learn from one another, or preferably with a health professional present to answer questions.

Participants felt that, although an mHealth short messaging system could be helpful for people who “understand how to use a mobile phone” or other digital devices, face-to-face groups would be more helpful for those who did not. Some participants also felt that face-to-face groups would be helpful for those who could not rely on their children to help them translate SMS text messages, noting that their children were often busy with their own lives. “Human contact” with health professionals and other patients was also seen as important, particularly in the context of receiving information:

I have come here twice and have informed others about what I learned here. They are all interested in joining. We can learn many things about the disease from which we are suffering. I am giving my opinion, like Mr X has said. When many people are involved in the discussion we can learn from them, like this son who has talked about many issues beautifully...If places are available, then I think it will be good for everyone...It will be better if a GP or doctor is present.

81-year-old Bangladeshi Muslim man

Other suggestions for disseminating message content included having educational programs or short adverts on South Asian television channels. Participants in the Bangladeshi Muslim men’s focus group stated that most Bangladeshi Muslim households in Leicester had audio receivers through which prayers and sermons from the local mosques were delivered. They felt that information about diabetes could be disseminated by faith leaders in local mosques and via the audio receivers to family members at home.

## Discussion

### Comparison With Prior Work

This is the first study exploring British South Asian views of a digital health intervention for patients with T2D. Our focus groups included a diverse sample, including the “seldom heard” views of people unable to speak in English, some of whom were also unable to write in English or a South Asian language.

Previous research into the perspectives of British South Asians with T2D has focused on experiences of, and barriers and facilitators to, DSM, but to date no research has explored the views of British South Asian people in relation to digital health interventions to support DSM. Our findings corroborate those of the HeLP-Diabetes study, guided by the Corbin and Strauss framework [60], where the features of digital health interventions desired by the general population included specific content relating to diabetes, reliable, accessible dietary advice, and guidance on emotional management [61]. Corbin and Strauss [60] discussed three types of work involved in living with and managing chronic illness: illness work, everyday life work, and biographical work. Illness-related work—that of managing symptoms, diagnosis, medications, and crises—differs from everyday life work, which includes managing everyday living, emotions, and relationships. Biographical work is the work that is done to find meaning from the condition and life experience in light of the disruptions to a person’s biographical narrative caused by chronic illness. For our participants, having messages that helped with illness-related work and everyday life work was of most importance in terms of DSM. Future DSM interventions aimed at South Asian populations could consider including culturally relevant information to support this “work” that people living with diabetes undertake in their everyday lives, such as information about the health benefits of different South Asian foods and diets, healthy portion sizes, fasting, and different kinds of physical activity.

The cultural adaptation of SMS text message interventions has generally included language translation, so that health messages reflect health beliefs, norms, and social practices [62]. However, there is little evidence to support the effectiveness and desirability of cultural adaptation of such interventions and a lack of research into the cultural appropriateness of messages [9]. Although a culturally adapted SMS has never been trialed in the United Kingdom, culturally adapted health promotion interventions aimed at British South Asians have shown only moderate effect and were inconclusive in demonstrating cost effectiveness [33]. None of these interventions involved head-to-head comparisons with interventions aimed at the general population. Study materials included adaptation into South Asian languages, which researchers state is challenging, time-consuming, costly, and not just a process of linguistic translation [63]. Participants in our study felt that messages delivered in South Asian languages were not essential or necessary for a short messaging intervention in which messages were less than 160 characters.

Previously adapted health promotion interventions for British South Asians with T2D have emphasized the importance of family involvement because of the strong cultural emphasis on family life. In a lifestyle intervention on weight change in British South Asian people at high risk of T2D, families rather than individuals were randomized in the trial. However, researchers working on the trial were unable to recruit family volunteers for many families, concluding that the added value of family involvement remains to be explored [64]. Our participants were positive about the involvement of family members or carers possibly because very little would be required of them to translate short messages. Recent work in intervention development recommends putting the needs and wants of end users at the heart of any intervention [50], highlighting that interventions that can be well integrated into everyday life and health care routines, that are easy to use, compatible with patients’ existing skills, and that do not significantly disrupt patients’ lives are more likely to lead to successful implementation [65]. Interventions aimed at South Asian populations may also need to consider these design aspects in relation to family and carers too.

A range of factors need to be considered when culturally adapting a digital health intervention, including levels of mobile phone or technology use. Access to smartphones can be influenced by various factors, including age, gender, education, and affordability [27]. It is also important to consider the power dynamics that determine different groups’ access to technologies [26] and how health inequalities may be created or perpetuated. A study of the use of electronic health among patients in Norway with type 1 and T2D found a strong association between a high level of education and the use of search engines but no educational differences for the use of apps, social media, and video services, indicating that adequate communication strategies for audiences with varying education levels should be a focus in efforts to reduce health inequalities in health outcomes [66]. As our participants suggested—users and nonusers alike—different digital formats for different groups have to be considered when thinking about the needs of heterogeneous populations with varied levels of literacy and education, as well as addressing the needs of family and community members who do not use digital devices, such as face-to-face meetings.

Our formative research sheds light on the varied needs of British South Asian patients in relation to short messages to support DSM. These findings are consistent with those from focus groups and interviews that we conducted separately with the general population, where participants also expressed a preference for message content that addressed all aspects of DSM and not just medication adherence [67]. This suggests that an mHealth SMS designed for the general population, such as the SuMMiT-D system, can be acceptable and relevant to UK South Asian populations but may need to include additional content with culturally adapted messages about South Asian foods, natural and herbal approaches used in the United Kingdom and South Asia, safe medicine taking when fasting, and exercise in women-only groups—messages that can be available to those who opt to receive them from a system that can provide individual choice. Other design implications worth considering include using images and audio messages for patients unable to read message content in English, as suggested by our participants. There is limited evidence of the extent to which culturally adapted messages might lead to specific changes in behavior, but they are likely to enhance engagement with the wider intervention. Exploring the views of British South Asian patients on an SMS aimed at the general UK population is important in maximizing the potential of such an intervention. Although head-to-head comparisons of adapted and nonadapted interventions are rare, our qualitative data with British South Asian communities and the general population suggest that formative work comparisons can yield helpful insights into which interventions need to be culturally adapted, why, and what this might involve. Future research should explore how best to codesign and test culturally adapted messages that could be incorporated into a general digital messaging system aimed at all UK patients with T2D.

### Limitations

The study was exploratory and, although it included some family/carer views, focus groups specifically with families would shed further light into their views on receiving short messages and translating these for patients. Although our sample included a diverse range of views, fewer second generation and no third generation participants took part in the study, people who may have had further ideas about system content and usability. As family/carers, third generation British South Asians could shed further light into the translating/explaining of message content to first- or second-generation family members (eg, to grandparents). As young patients, they may have differing views about the need for messages on, for example, South Asian foods and diets. As regular users of the internet, they may also feel that they have access to all the information that they need and have no need for short digital messages. The focus groups were held during the day. Evening focus groups might have encouraged more second and third generation people to attend, potentially a more convenient time for those in full-time education and employment.

### Conclusions

An mHealth short messaging intervention that addresses all aspects of DSM is more relevant and acceptable to British South Asian people with T2D than one that focuses only on medication adherence. For such an intervention to meet the needs of UK South Asian populations, it may also have to include culturally relevant messages sent to those who opt to receive them. It is equally important to consider how to disseminate message content to patients who do not use digital devices to help reduce health inequalities, including face-to-face groups.

## Appendix

Multimedia Appendix 1 Participants’ quotes about system content and usability.

## Abbreviations

CBMEH: Centre for Black and Minority Ethnic Health
DSM: diabetes self-management
GP: general practitioner
mHealth: mobile health
NIHR: National Institute for Health Research
SuMMiT-D: Support Through Mobile Messaging and Digital Health Technology for Diabetes
T2D: type 2 diabetes
