# Supplementary material for: British South Asian Patients’ Perspectives on the Relevance and Acceptability of Mobile Health Text Messaging to Support Medication Adherence for Type 2 Diabetes: Qualitative Study
Source: JMIR Mhealth Uhealth. 2020 Apr 20;8(4):e15789. doi: 10.2196/15789 (PMC7199132; doi:10.2196/15789)
Supplement: Multimedia Appendix 1 [file mhealth_v8i4e15789_app1.docx]

**Appendix 1. Participant quotes about a digital health brief messaging system to support diabetes medication adherence**

**Message content and design features**

*What I feel is important is we get something in terms of our symptoms, guidance, in the sense that this is the, this is the research that has been done recently, and this is what...so you get fresh messages, not messages that will be repetitive all the time.*

*[69-year-old Gujarati Hindu man]*

*M1 What other messages would we want?*

*F1 The quantity of food we should eat, like how much in the morning, how much in the afternoon.*

*F3 During Ramadan month, taking medicine is difficult for us, we have our meal after performing Iftar, messages on this subject will be helpful....During Ramadan month, if we have to take medicine, how we should take them?*

*[M1: 47-year-old Bangladeshi Muslim man; F1: 34-year-old Bangladeshi Muslim woman; F3: 41-year-old Bangladeshi Muslim woman]*

*The last message on the second page is: ‘There are some medicines known as homeopathic medicine that will help your diabetes tablet to suppress your diabetes. Check with your doctor.’ How beneficial will this type of message be? Like you talked about fenugreek, onion seeds, these types of spices. Now these may be harmful for you, but how will you know that?....This message is quite important. Because quite a few of these people are taking alternative medications and very often, well, because somebody else has asked them to take it and they apply it.*

*[47-year-old Bangladeshi Muslim man]*

*Reminders regarding your tests, especially blood tests, you know, when it's due, you know, just a reminder because it's not due every day, or it's not due every month. It's usually six months, yearly or something…if it's yearly just send them a message saying.....'Look your blood test is due,' which is very vital, and a lot of people forget, especially elderly people. I forget, you know.*

*[58-year-old Gujarati Hindu man]*

*The other thing is, if they're going to advise about exercise, if you are talking about that as well, then people who cannot walk, or have limited walking abilities, what should they be doing?....That is very useful, even if it's daily, it's not harmful, but this [messages about exercise] twice a week I think we should get it. They're advising doing it [exercise] every day now, isn't it?*

*[69-year-old Gujarati Hindu man]*

*What about food, you know? Because do we know how much is a good portion? How much is a good portion? Do we know how much is good? How much should we eat and how much is too much? Do you have any understanding of this?...[...]...I eat food which seems appetizing and delicious. I even eat when I am full. As I am a diabetic, it would be helpful if there was something to remind me when to eat and how much to eat.....What if text messages about a balanced diet are sent to us? Then we won't be able to deny it. What is a balanced diet? It could show us the amount of carbohydrate, fat, glucose, and protein in each food. Sometimes we don't bother thinking about that and eat emotionally.*

*[47-year-old Bangladeshi Muslim man]*

**Language preferences**

*I mean for people that don’t know how to kind of read English, it would probably be beneficial for them to get it in the language that they speak or read, because I know some people can't probably get anyone to translate for them every time, [um] so I think yeah, it would be a good idea if they can do it in different languages. But obviously most of the people do understand English.*

*[36-year-old Bangladeshi Muslim woman]*

*F2 English is good....*

*F1 Because it’s a different language, like Urdu – the written Urdu is hard [to understand, more formal], that’s why English is better.*

*F2 The words are hard, that’s why. I try to read Urdu but it’s no good, it’s a different Urdu, so....*

*F1 It’s hard maybe...it’s not always separate words so we, it’s joined, so it’s very hard. So English is good.*

*[F1: 46-year-old Pakistani Muslim woman; F2: 31-year-old Bangladeshi Muslim woman]*

**Family involvement**

*I think nowadays that everybody has a smartphone, mobile phone, so I wouldn’t say it's that, [um] it's just a matter of understanding it. But most people can get their ways around it because they’ve got family members, they’ve got children, they can usually just get them to translate for them, so I don’t think it's really a big problem. Maybe only for very odd few people it would be.*

*[36-year-old Bangladeshi Muslim woman]*

*It is such a practical problem. Often in my house, when the women have finished cooking, they want to feed us and then they want to finish eating. It does not matter whether you are hungry or not; you have to eat according to their timetable. I am sure that this problem prevails not only in my house, but also in others. So, I think this matter should be given some thought. We should consider sending these messages not only to you, but also to your family members.*

*[47-year-old Bangladeshi Muslim man]*

**Different digital formats for different groups**

*There's a huge difference between the generations obviously, so technology, at the end of the day, if I said things like Snapchat and Twitter here, most of the people, with all due respect, wouldn't know what I'm talking about here. So obviously, the younger generation, I mean I'm technology-minded but I don't use Snapchat, but I know my kids do. And the younger generation use it. And they are really hot on that. So there is obviously generation difference. What the younger people are going to be able to access is different to what even myself now, and certainly the older generations here, will access. So you do have to have different mediums.*

*[46-year-old Bangladeshi Muslim man]*

*SP If someone didn’t read English, what could be done to help them?*

*F1 Voice messages.*

*F2 Voice messages are good in that case, for those who don’t understand....I think this is good. A second option could be voice messages.*

*SP A second option is voice messages. The first option would be?*

*F1 Written messages.*

*[SP: Interviewer; F1: 46-year-old Pakistani Muslim woman; F2: 31-year-old Bangladeshi Muslim woman]*

**Face-to-face groups for those who do not use digital technology**

*FP3: That [text messages] would help to some extent for those people who understand how to use a mobile, how to use a laptop, how to use a tablet. There are many people frankly who doesn’t know how to use it..... Many people who are out there who don’t know how to read and write; they don’t know how to use a mobile; they don’t how to use a laptop. So then what should be done? But we have to think about them, you know. There are many people who don’t know how to use mobile phones.... The older generation, they are not [um] text savvy.... I think first of all I'd give them a little bit more time, just you know, just face to face with them, you know.*

*[50-year-old Pakistani Muslim woman]*

*Face to face is good for people who are not into apps and into technology. I think it's very good to meet socially and in places like this and local Masjids[Mosques] or community centres. It’s very good.....There is a lack of knowledge amongst people so all of these means like the technology and whatever, is the means to get that information. But for specific age groups and specific types of people, you need to find different avenues. Yeah. So for this particular age group, I think meetings and gatherings like this are going to be very, very beneficial, where they can share and maybe with the presence of medical professionals.*

*[46-year-old Bangladeshi Muslim man]*

*So, the idea is here, from this gentleman here, is that everybody goes to the Masjid. Especially elderly people, but on a Friday, everybody goes. So if the Imam-sahib could deliver in his sermon talks in matters related to this, then that would be very useful. So I posed the question, but in the Masjid only the men folks go. What about the women folks? And the answer that came from the floor was that we all have receivers in our homes, which is connected to our local Masjids, so the women folk could actually get the messages through the radio in the home. So it would be a good way of conveying this message to the, certainly the Muslim community.*

*[47-year-old Bangladeshi Muslim man]*

*You can use the mosque as a means of reaching the people because a significant portion of the population are Muslims. And if the information is given, under controlled conditions, through the Imams, people can be reached. If you just give them the information, you ought to explain to them, and all these views in many of the Mosques and there are very many Bengali Mosques. They will then have that level of information. This is an effective way of reaching the people....There are some Bengali channels, you know, where there you can have reached the people, you know. But people watch Bengali channel. I watch it almost every day for many years. [71-year-old Bangladeshi Muslim man]*
